# Chloroform Inhalations and Injections of Air in Ileus

**Published:** 1853-09

**Authors:** 


*Chloroform Inhalations and Injections of Air in Ileus.* By MERZ.—A girl, aged 27, up to the present time in good health, was suddenly seized with stercoraceous vomiting without obvious cause. After narcotics and mild drastic purgatives had been tried without avail, injections of air per anum were administered, with the effect of mitigating her distress. Then chloroform was inhaled so as to produce complete paralysis, when a rumbling or rolling of the bowels ensued, followed by discharge of fæcal-smelling flatus and copious evacuations; the patient recovered.—*Med. Times and Gaz.*, from *Schw. C. Ztschr.*, 1853.

